# Clinical Lecture in Mercy Hospital

**Published:** 1861-07

**Authors:** N. S. Davis

**Affiliations:** Prof. of Clinical Med., etc.


*Clinical Lecture in Mercy Hospital, April* 2, 1861, *on Chronic Gastritis, Post-Mortem Appearance, Inflammation of the Kidneys, etc.* By N. S. Davis, M. D., Prof. of Clinical Med., etc.

Gentlemen:—At the last clinic hour, your attention was occupied chiefly with a case of chronic inflammation of the mucous membrane of the stomach, coupled with indications of incipient tuberculosis. We then not only stated the symptoms of the disease as, illustrated in the patient brought before you, but we alluded also, briefly, to the diagnosis between it and the different varieties of cancer on the one hand, and mere functional derangement of the stomach, on the other. We reminded you that chronic gastritis is generally characterized by a distinct burning, smarting pain, increased by food; frequently the rejection of the latter by vomiting, in a sour or acrid condition, or mixed with mucous; acceleration of the pulse; some tenderness of the epigastrium; the tongue red, sometimes smooth and glossy on its surface, at others fissured and tender; the bowels generally inactive though sometimes relaxed, with apthous sores in the mouth. The urine is in most cases scanty and high colored, and the emaciation progressive. We reminded you that these symptoms differed from those of simple functional derangements of the stomach in several respects. They are more constant or uniform from day to day, while functional derangement is seldom accompanied by either redness of the tongue, acceleration of the pulse, or any considerable emaciation; and if tenderness of the epigastrium exists, it is only for a day at a time, while the distress from food is rather a load or weight, with gaseous eructations, instead of burning, smarting and vomiting, as in inflammation. The differential diagnosis between chronic inflammation of the stomach and cancer, was admitted to be more obscure and difficult to define; so difficult indeed that the experienced are sometimes left in doubt. We reminded you, however, that the symptoms of cancer usually commence much more gradually and obscurely than those of chronic inflammation.

There is for a long time less alteration of the pulse, less redness of the tongue, less thirst, while the emaciation is accompanied by that mixture of the sollow and anæmic hue of the skin peculiar to the cancer cachexia; and the abdomen is almost constantly sunken or empty, and the bowels costive. We have briefly recalled these symptoms, which were dwelt upon much more minutely in the preceding lecture, for the purpose of introducing to your notice some pathological specimens obtained by a recent post-mortem examination. We were called directly from the clinic, to which we have alluded, to the bed-side of a patient whom we found dying. He had the appearance of one decidedly anæmic, but was only moderately emaciated, with some anasarcous swelling of the extremities.

His pulse was feeble and thread-like, extremities cold, breathing oppressed, in fact he seemed apparently in articulo mortis.

From his wife and friends we learned that about one year since he had an attack of fever, and, during his treatment, he was severely salivated with mercurials. His general health had not been good since that time. He had been constantly more or less anæmic and troubled with indigestion. Still he had continued to attend to his ordinary duties until about six weeks since, when his food began to give him more distress, and much of it was rejected by vomiting. His strength, of course, rapidly failed, and during the last three weeks he retained neither food nor drink. He had some pains in his back and head, and œdema of the extremities. The condition of the urine could not be ascertained, as no attention had been paid to that subject. The medical attendant, who was not a regular member of the profession, had pronounced the disease to be *cancer* of the stomach. But its evident beginning after the attack of fever and salivation, the persistent and active vomiting during the last three weeks, together with the œdema of the extremities and paroxysms of headache, led us to doubt the existence of cancer, and to express the opinion that the disease was chronic gastritis, complicated with some morbid condition of the kidneys. In about an hour after this visit the patient died, and the next day we were requested to make a post-mortem examination. We complied with this request, assisted by Dr. M. O. Heydock, of this city. The cavity of the abdomen was opened in the usual manner, and its viscera carefully examined. The liver was perfectly natural in size, color and structure, and the gall bladder was moderately full of yellew bile.

The spleen was natural in color, but about one-third larger than the normal size, unusually firm and dense in its structure, and on its outer or costal surface there was a spot about one inch in diameter, where, on cutting into it, the investing membrane was found to contain a true bony deposit, so firm as to resist the scalpel. Here is a portion of that part of the spleen, and by passing it from one to the other, each of you can see the change of structure, and you will observe also that the hard or bony deposit is limited strictly to the surface. The whole exterior of the intestines and mesentery appeared healthy. The only evidences of disease found in these organs were in the mucous membrane of the stomach, which is here exhibited for your examination. That part lining the lesser curvature of the stomach is intensely red, with here and there a dark spot, moderately thickened; and on close examination two or three spots will be seen abraded or deprived of epithelium. An unnatural degree of redness is also seen over that part lining the left portion and larger curvature of the stomach, but less than in the part just described.

As we approach the pylorus the membrane appears more natural, and the pylorus itself is perfectly healthy.

Thus, all the morbid appearances in the stomach are such as indicate the existence of simple chronic or sub-acute inflammation of the mucous membrane, without the least vestige of malignant or cancerous disease. The bladder was found moderately distended with urine, and the left kidney entirely natural. On lifting the right kidney from its place, it was found slightly larger and darker red than the left; and on laying it open, its whole texture was intensely injected with blood, forming a strong contrast when compared with the other. The specimen is here before you, and though faded some by maceration during the last forty-eight hours, yet the morbid redness is still easily recognized. The further progress of the post-mortem revealed no other evidences of disease. It is thus seen that the opinion expressed by the attending physician, that the patient was laboring under cancer of the stomach, was entirely erroneous.

The morbid condition of the spleen had undoubtedly existed a considerable length of time; probably since the attack of fever one year since. As the function of the spleen evidently exerts some influence, either direct or indirect, over the formation of red-corpuscles of the blood, its morbid condition might explain the anæmic appearance exhibited by the patient during the last six or eight months. This was also accompanied by sufficient gastric derangement to occasion imperfect and sometimes painful digestion. These embarrassments, however, were not sufficient to prevent him from attending to his duties in an active out-door occupation until four or five weeks since, when the active and persistent vomiting commenced and continued until death. This symptom doubtless marked the commencement of the inflammation which you have seen in the mucous membrane of the stomach, and the parenchyma of the right kidney. Whether the inflammation in these localities commenced simultaneously, from the same general causes or not, cannot be determined with certainty by the post-mortem appearances. But I wish to remind you that there is a close relation in many cases between a morbid condition of the kidneys and gastric irritation. A case came under our care a year or two since, in which the urinary secretion was very scanty, amounting to not more than four ounces in the twenty-four hours, and highly albuminous, with general anasarca, and a profuse watery diarrhœa. These symptoms had supervened during the period of convalescence from an attack of remittent fever.

Fearing the occurrence of extreme exhaustion from the continuance of diarrhœa, remedies were administered for the purpose of restraining it, and at the same time favoring the increased secretion of urine. But it was soon found that whenever the intestinal discharges were restrained for twenty-four hours, either active vomiting ensued or symptoms of approaching coma. This led to the suspicion that the diarrhœa was purely vicarious or the result of uœmic irritation; and on applying the tests, urea was readily detected in the intestinal discharges. The patient ultimately recovered under the influence of treatment for inflammatory congestion of the kidneys.

If you make the necessary inquiries you will find many cases in practice, illustrating the effects of urinary disorder on the gastric and cerebral functions. It is now well known that a large proportion of the convulsive affections, not only of puerperal women, but of children also, arise from the retention of urea in the blood.

We have found many cases of that distressing affection called "sick headache," which had recurred again and again for a long period of time, permanently relieved by establishing and maintaining a full healthy action of the kidneys. In the case immediately before us, whether the inflammation of the kidney preceded that of the stomach, or merely accompanied, there can be no doubt but that it contributed much to increase the gastric irritability and advance the patient towards a fatal result. Yet its existence appears not to have been suspected during life. Such cases should admonish you to acquire the habit of examining all patients carefully in reference to every important function, instead of allowing the attention to be wholly engrossed with the more prominent symptoms, as is too often the case.

We shall allow you to occupy the remainder of the present clinic hour in examining the physical signs of pneumonia complicating a case of extensive tuberculosis.—*Medical Examiner.*

